# HPV16 Oncoproteins Induce MMPs/RECK-TIMP-2 Imbalance in Primary Keratinocytes: Possible Implications in Cervical Carcinogenesis

**DOI:** 10.1371/journal.pone.0033585

**Published:** 2012-03-16

**Authors:** Laura Beatriz da Silva Cardeal, Enrique Boccardo, Lara Termini, Tatiana Rabachini, Maria Antonieta Andreoli, Celso di Loreto, Adhemar Longatto Filho, Luisa Lina Villa, Silvya Stuchi Maria-Engler

**Affiliations:** 1 Clinical Chemistry and Toxicology Department, School of Pharmaceutical Sciences, University of São Paulo, São Paulo, Brazil; 2 Ludwig Institute for Cancer Research, Virology Group, São Paulo, Brazil; 3 Department of Microbiology, Institute of Biomedical Sciences, University of São Paulo, São Paulo, Brazil; 4 HPV Institute – INCT-HPV, Santa Casa de Misericórdia, São Paulo, Brazil; 5 Nucleo de Patologia do Instituto Adolfo Lutz, São Paulo, Brazil; 6 Laboratory of Medical Investigation (LIM) 14, School of Medicine, University of São Paulo, São Paulo, Brazil; 7 PIO XII Foundation, Barretos Cancer Hospital, Barretos, Brazil; 8 Life and Health Sciences Research Institute (ICVS), School of Health Sciences, University of Minho, Braga, Portugal; 9 ICVS/3B's - PT Government Associate Laboratory, Braga/Guimarães, Portugal; IPO, Inst Port Oncology, Portugal

## Abstract

Cervical cancer is the third most common cancer in women worldwide. Persistent infection with high-risk HPV types, principally HPV16 and 18 is the main risk factor for the development of this malignancy. However, the onset of invasive tumor occurs many years after initial exposure in a minority of infected women. This suggests that other factors beyond viral infection are necessary for tumor establishment and progression. Tumor progression is characterized by an increase in secretion and activation of matrix metalloproteinases (MMPs) produced by either the tumor cells themselves or tumor-associated fibroblasts or macrophages. Increased MMPs expression, including MMP-2, MMP-9 and MT1-MMP, has been observed during cervical carcinoma progression. These proteins have been associated with degradation of ECM components, tumor invasion, metastasis and recurrence. However, few studies have evaluated the interplay between HPV infection and the expression and activity of MMPs and their regulators in cervical cancer. We analyzed the effect of HPV16 oncoproteins on the expression and activity of MMP-2, MMP-9, MT1-MMP, and their inhibitors TIMP-2 and RECK in cultures of human keratinocytes. We observed that E7 expression is associated with increased pro-MMP-9 activity in the epithelial component of organotypic cultures, while E6 and E7 oncoproteins co-expression down-regulates RECK and TIMP-2 levels in organotypic and monolayers cultures. Finally, a study conducted in human cervical tissues showed a decrease in RECK expression levels in precancer and cancer lesions. Our results indicate that HPV oncoproteins promote MMPs/RECK-TIMP-2 imbalance which may be involved in HPV-associated lesions outcome.

## Introduction

Cervical cancer is the third most commonly diagnosed malignancy and the fourth leading cause of cancer death in females worldwide. It accounts for 9% (529,800) of the total new cancer cases and 8% (275,100) of the total cancer deaths among females [1]. Molecular and epidemiological studies have established that persistent infection with high-risk human papillomaviruses (HR-HPVs) types is the main risk factor for the development of cervical cancer and its precursor lesions [2], [3]. HPVs are small DNA viruses that encode two viral oncoproteins, E6 and E7. The best characterized properties of E6 and E7 proteins from HR-HPVs are their capacity to mediate the degradation of p53 and pRb, respectively. Besides, these viral proteins interact with other several cellular factors abrogating normal cell cycle checkpoints and cell death mechanisms [4], [5]. E6 and E7 are essential for establishment and progression of cervical cancer precursor lesions being the only viral products consistently expressed in cervical cancer derived cell lines. Importantly, their continuous expression is required to maintain the transformed phenotype *in vitro* [6], [7].

Progression of most solid tumors is characterized by an increase in secretion and activation of matrix metalloproteinases (MMPs) produced by either the tumor cells or tumor-associated fibroblasts [8], [9]. These proteins play a central role in diverse physiological processes and diseases such as homeostatic tissue remodeling and cancer. Proteolytic activity of MMPs can be regulated at different levels including gene expression, protein compartmentalization, zymogen to active enzyme conversion and by the presence of specific inhibitors [10], [11]. Tissue or extracellular MMPs are regulated by endogenous inhibitors named tissue inhibitor of metalloproteinases 1 to 4 (TIMP-1–4). Among them, TIMP-2 is unique since it may function both as a MMP inhibitor and activator [9]. Besides, REversion-inducing Cysteine-rich protein with Kazal motifs (RECK), a membrane bound protein, can exert inhibitory effects on the transcription, synthesis, activation and activity of MMPs [12], [13], [14]. RECK gene is expressed in various normal organs and has been found to be important in suppressing tumor invasion, metastasis and angiogenesis [12], [13], [15]. Several studies have shown that RECK levels are significantly down-regulated in human pancreatic, mammary, lung, prostate, and colorectal cancers, as well as in hepatocarcinoma and neuroblastoma, compared with the normal surrounding tissue [16]–[22]. Importantly, detection of normal or elevated RECK levels in tumor samples has been associated with decreased invasiveness and metastatic potential and with improved prognosis as well [23].

Increased MMPs expression, including MMP-2, MMP-9 and MT1-MMP, has been observed during cervical carcinoma progression. These proteins have been detected in tumor and peritumoral stroma cells and associated with degradation of extracellular matrix (ECM) components. Moreover, their involvement in tumor invasion process, metastasis and tumor recurrence has been described [24]–[28]. However, few studies have evaluated the interplay between HPV infection and the expression and activity of different MMPs and their regulators in cervical carcinogenesis [29], [30].

In the present study we analyzed the effect of HPV16 oncoproteins on the expression and activity of MMP-2, MMP-9, MT1-MMP, TIMP-2 and RECK in monolayer and organotypic cultures of human keratinocytes. We observed that E7 expression is associated with increased pro-MMP-9 activity in the epithelial component of organotypic cultures. Besides, we show that E6 and E7 co-expression down-regulates RECK and TIMP-2 levels in organotypic and monolayers cultures. Interestingly, immunohistochemical analysis in human cervical tissues show a decrease of RECK levels in precancer and cancer lesions. Altogether, our results support the notion that HPV infection, through the expression of E6 and E7, promotes MMPs/RECK-TIMP-2 imbalance that may affect HPV-associated lesions outcome.

## Materials and Methods

### Cell lines and Retrovirus derived Vectors

Human cervical carcinoma-derived cell lines (SiHa, CaSki, C33A), human fibrosarcoma-derived cell line (HT1080) and murine fibroblasts J2 (NIH 3T3, ATCC: CRL1888) were obtained from the American Type Culture Collection (Manassas, VA, USA). The primary culture of human fibroblasts (FF 287) was a gift from Dr. María Soengas (Centro Nacional de Investigaciones Oncológicas CNIO / Spanish National Cancer Research Center, Madrid, Spain). Cell cultures were maintained in DMEM supplemented with 10% FBS. Low passage-pooled neonatal human foreskin keratinocytes (HFK) (Lonza Walkersville, Inc., Walkersville, MD, USA) were cultured in keratinocytes serum-free medium (KSFM, Invitrogen, Frederick, MD, USA).

pLXSN-based retroviral vectors (access Number M28248, GeneBank) containing HPV16 E6 and/or E7 wild type sequences were a kind gift from Dr. Denise A. Galloway (Division of Human Biology, Fred Hutchinson Cancer Research Center, Seattle, WA, USA). Human foreskin keratinocytes were infected with retroviral particles containing pLXSN vector alone or HPV16 E6 and/or E7 oncogenes. After 24 hours, the cells were selected with 300 µg/ml of G418 for 2 days. Surviving cells were amplified and used to seed organotypic or monolayers cultures.

### Organotypic raft cultures

pLXSN, E6wt, E7wt and E6E7 transduced HFK cell lines were differentiated in organotypic raft cultures for 9–11 days at the medium-air interface as described previously [31]. The cultures were harvested, fixed in 10% buffered formalin, embedded in paraffin, and cut into 4-µm histological sections.

### Tissue samples

Cervical tissue samples were obtained from the Laboratory of Medical Investigation (LIM) 14, Department of Pathology, São Paulo University School of Medicine, Brazil, and from the Pathology Division, Adolfo Lutz Institute, São Paulo, Brazil. Ethical approval for this study was granted by the Comitê de Ética em Pesquisa do Instituto Adolfo Lutz (CEPIAL – Research Ethical Committee of Adolfo Lutz Institute). There was no informed consent because tissue samples were selected retrospectively from a routine pathology archive, in a completely anonymous way. All the samples were older than three years. Therefore, it was not possible to track back written informed consent of the patients. A total of 17 paraffin-embedded cervical tissue specimens and 49 TMA (Tissue Micro Array) were analyzed for RECK staining. These samples included 16 cervicitis, 20 cervical intraepithelial neoplasias grade I (CIN I), 18 CIN II/III and 12 invasive carcinomas.

### Western Blot analysis and gelatin Zymography

After 11 days at the liquid–air interface, epidermis from raft cultures were detached from the collagen matrix (dermal equivalent) with a scalpel and frozen in liquid nitrogen. Epithelial tissues from two cultures were minced in a tissue grinder containing 200 µl of cold lysis buffer: 50 mM Tris pH 7.4, 150 mM NaCl, 1% Triton X-100, 1 mM EDTA, and proteases inhibitors (Protease inhibitor cocktail tablets; Roche, Mannheim, Germany). After 15 min on ice, the tubes were centrifuged for 20 min at 10000× *g* and 4°C to separate the insoluble material. Cell lines and transduced keratinocytes grown in monolayer were lysed in the same cold lysis buffer. Protein concentrations were determined by a commercial protein assay (Bio-Rad Laboratories, Hercules, CA, USA). Equivalent amounts of protein (25 µg for MMPs blots and 50 µg for RECK blots) were resolved by SDS-PAGE (8–10%) and transferred to polyvinylidene difluoride membranes (Amersham Pharmacia Biotech, Buckinghamshire, UK) using the Mini Trans-blot Electrophoretic Transfer system (Bio-Rad Laboratories, Hercules, CA, USA). Membranes were blocked for 1 h in 5% non-fat milk and probed overnight with the following primary antibodies: anti-MMP-2 (MAB3308) at 1∶1000, anti-MMP-9 (MAB13415) at 1∶100, anti-MT1-MMP (AB8345) at 1∶250, all three from Chemicon (Temecula, CA, USA); anti-RECK (611512,BD Biosciences; San Jose, CA, USA) at 1∶250. Expression of β-actin and α-tubulin was used as a loading control and detected with anti-actin (a5441) and anti-tubulin (t5168) both from Sigma (St Louis, MO, USA). Membranes were washed and then reprobed with horseradish peroxidase (HRP)-conjugated secondary antibodies. The bands were revealed using Enhanced Chemiluminescence procedures according to the manufacturer's recommendations (Amersham Pharmacia Biotech, Buckinghamshire, UK). Samples from HT1080 (human fibrosarcoma) and FF287 (human fibroblast) were used as positive controls for MMPs and RECK, respectively.

Gelatinolytic activity was examined by gelatin zymography. Equal amounts of total proteins extracts from rafts epidermis and dermis were separated on 10% SDS-PAGE gels containing 1 mg/ml gelatin. The gels were washed twice in 2.5% Triton X-100 at 37°C and incubated overnight at 37°C in reaction buffer (0.05 M TrisHCl, pH = 8.5, 10 mM CaCl_2_, 1 µM ZnCl_2_). Gels were submitted to stain with coomassie solution (0.5% Coomassie brilliant blue R-250, in 10% methanol, and 10% acetic acid) and destained in 10% methanol/10% acetic acid. Clear zones of gelatin lysis against a blue background stain indicated the presence of MMPs. Each lysis zone within a given sample lane was analyzed using Image J software (National Institute of Health, Bethesda, Maryland, USA).

### Measurement of MMP-2 and MMP-9 activity

MMP-2 and MMP-9 activity was quantified in the culture supernatants from monolayer cultures of HFK transduced with retroviral vectors expressing HPV16 oncoproteins using specific Biotrak assay systems (MMP-2 Biotrak Activity Assay RPN 2631; MMP-9 Biotrak Activity Assay RPN2634, GE Healthcare, Buckinghamshire, UK) according to the manufacturer's instructions. One hundred micrograms of total protein from the corresponding culture medium was added to each well of the Biotrak plates. The absorbance was measured by spectrophotometry at 405 nm. MMP-2 and MMP-9 activities were calculated using standards provided by the kit and expressed as ng/ml. The detection limits of the assay are 0.75–12 ng/ml for MMP-2 and 0.125–16 ng/ml for MMP-9. Each cell line was analyzed in triplicate.

### Immunofluorescence

Transduced HFK were plated on glass coverslips, fixed with 4% paraphormaldehyde in phosphate-buffered saline (PBS), washed with PBS, and permeabilized with 0.2% Triton X-100 in PBS for 5 min at room temperature (RT). Cells were then blocked with 5% bovine serum albumin in PBS at RT for 30 min and incubated for one hour with anti-RECK (BD Biosciences, San Jose, CA, USA) at 1∶50 dilution in blocking solution at RT. An Alexa Fluor 488-conjugated goat anti-mouse antibody was used as secondary antibody (1∶200; A-11001, Molecular Probes, CA, USA). Nuclei were counterstained with 4′,6-diamino-2-phenylindole (DAPI, Molecular Probes, CA, USA). Sections were mounted with ProLong Antifade Kit (P7481, Molecular Probes, CA, USA). Images were captured with a Nikon Eclipse S100 fluorescence microscope, with Metamorph (6.0, Molecular Devices, PA, USA) software (DAPI: 100 ms and Alexa 488: 600 ms). The primary antibody was omitted in the preparation of negative control coverslips.

### TIMP-2 ELISA assay

Culture supernatants obtained as described before were examined for expression of TIMP-2 using a sandwich enzyme-linked immunosorbent assay (TIMP-2, Human, Biotrak ELISA System RPN 2618, GE Healthcare, Buckinghamshire, UK) according to the manufacturer's instructions. Briefly, 100 µg of total protein from the corresponding culture medium or the supplied standards were incubated with a peroxidase labeled Fab′ antibody to TIMP-2. Immediately, samples were added to microtitre wells pre-coated with anti-TIMP-2 antibody and incubated for 2 h at room temperature. The wells were rinsed with washing solution, and TMB substrate (tetramethylbenzidine) was added. The reaction was stopped by the addition of 1 M sulfuric acid. The absorbance of each well was measured by spectrophotometry at 450 nm and plotted against a standard curve with TIMP-2 levels expressed as ng/ml. The lower detection limit of this assay is 5.0 ng/ml. Each cell line was analyzed in triplicate.

### Immunohistochemical staining of organotypic cultures and tissue samples

Paraffin-embedded organotypic cultures and tissue samples sections were deparaffinized in xylene and rehydrated in alcohol. After antigen retrieval with citrate buffer (pH 6.0) sections were incubated with 3% hydrogen peroxidase in PBS to block endogenous peroxidase activity. Tissue samples were additionally blocked with 10% horse serum (Gibco - #16050130, Carlsbad, CA,USA). Sections were incubated overnight at 4°C with an anti-RECK monoclonal antibody (611512 / BD Biosciences; San Jose, CA, USA) at 1∶100 in 2% bovine serum albumin-phosphate buffered solution for organotypic cultures, or at 1∶75 in 5% horse serum phosphate buffered solution for cervical tissue samples. After washing in PBS, the organotypic cultures sections were incubated with the LSAB+ System-HRP –K0690 kit (DAKO Cytomation, CA, USA) and cervical tissue samples with Histostain-SP broad spectrum secondary antibody (Zymed Laboratories, South San Francisco, USA), according to the manufacturer's instructions. Slides were developed using 3,3′-diaminobenzidine tetrahydrochloride (Dako Cytomation Liquid DAB+substrate Chromogen System Kit, K-3467, Dako North America, CA, USA, or D-5637, Sigma, USA) and counterstained with Harry's hematoxylin. An irrelevant mouse IgG was used in the preparation of negative control slides. Human skin samples were used to prepare positive control slides. In cervical tissue samples RECK-expression was estimated as follows: Grade 0, no staining; Grade 1, faint staining; Grade 2, moderate staining; Grade 3, strong staining, for membrane and cytoplasmic detection. To facilitate the analyses, tumors were classified based on the intensity and localization of the staining, into the following three groups: RECK-weak tumors (Grades 0–1 at membrane), RECK-strong tumors (Grades 2–3 at membrane) and RECK cytoplasmic (Grades 1, 2 and 3 at cytoplasm). The images were captured with an Olympix DP70 digital camera attached to an Olympus Provis IX70 microscope with Image-Pro Plus 5.0 software.

### Quantitative real-time RT-PCR (qRT-PCR)

Total RNA was isolated from transduced keratinocytes with RNeasy MiniKit (Qiagen, Valencia, CA, USA), according to the manufacturers protocol. RNA was quantified spectrophotometrically (ND-1000 – NanoDrop- Wilmington, DE, USA). One microgram of total RNA, previously treated with 1unit/µL DNAse I, was reverse transcribed using 0.5 µg/µL of oligo-dT primers, 100 ng/µL of random hexamers and 200 units/µL of M-MuLVRT reverse transcriptase (Fermentas, Glen Burnie, MD, USA) and the resulting cDNA was diluted 120-fold. Specific primers for human RECK: F: 5′- TGCAAGCAGGCATCTTCAAA - 3′ and R: 5′- ACCGAGCCCATTTCATTTCTG - 3′, and Tubulin: F: 5′- TCAACACCTTCTTCAGTGAAACG - 3′ and R: 5′- AGTGCCAGTGCGAACTTCATC - 3′, were designed using Primer3 software and synthesized by IDT Inc, (Coralville, IA, USA). Tubulin was used to normalize the amount of total human cDNA. Reactions were carried out in a 7500 Real-Time PCR System (PE - Applied Biosystems, Scoresby, Australia). The PCR conditions and relative gene expression analysis were described previously [29], [32].

### Statistical Analysis

Statistical analysis was performed with GraphPad Prism 5 (GraphPad Software, Inc., CA, USA). The significance of detected expression or activities differences was calculated by One-way ANOVA followed by a multiple comparison test (Tukey test). All the experiments were performed at least three times, and the total number of experiments is indicated in the figure legend. Data are expressed as mean ± SE. Statistical significance was defined as *P*<0.05.

## Results

### MMP-9 activity is up-regulated in organotypic cultures expressing HPV16 E7

We have previously shown that expression of MMP-9 and MT1-MMP is higher in HPV positive than in HPV negative cervical cancer derived cell lines [29]. This prompted us to analyze the individual and combined effect of HPV oncoproteins on the level and activity of MMP-2, MMP-9 and MT1-MMP in HFKs grown in monolayer. We observed that HPV16 oncoproteins do not affect the level or activity of these MMPs in monolayer cultures of HFKs (Figure S1).

**Figure 1:**
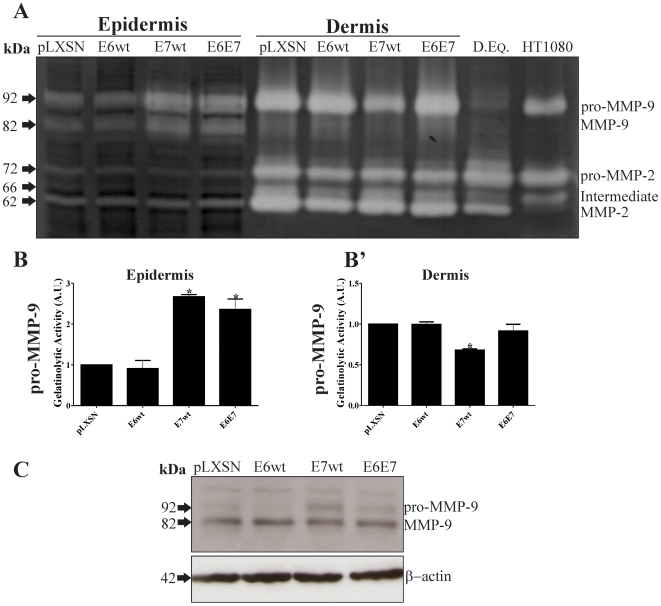
HPV-16 E7 up-regulates MMP-9 activity in organotypic cultures. Primary HFKs were transduced with pLXSN-based retroviral vectors expressing HPV16 E6wt and/or E7wt. Cells were then differentiated in organotypic cultures for 9 to 11 days. *A,* Determination of gelatinase activity in organotypic cultures homogenates (epidermis separated from dermis). Equal amounts of proteins were loaded. Note the increase in MMP-9 gelatinase activity in HPV-16 E7wt and E6E7- expressing epidermis. *B-B′,* densitometry of pro-MMP-9 bands in epidermis and dermis, respectively. *C,* MMP-9 levels in epidermis homogenates were determined by Western blot. *P<0.05*.

HPV life cycle is tightly associated to stratified epithelial differentiation and many effects of viral oncoproteins take place in the suprabasal layers of squamous epithelia. Therefore, we investigated the impact of viral oncoproteins on the expression of MMP-2, MMP-9 and MT1-MMP in epithelial organotypic cultures. The relative amounts and activity of latent and activated MMP-9 and MMP-2 molecular species were assessed by western blot and gelatin zimography, respectively, in of epidermis (stratified keratinocytes) or dermis (fibroblast and type I collagen gel) from organotypic cultures expressing HPV16 oncoproteins (Figure 1). Gelatinolytic activity was detected in bands around 92 kDa (pro-MMP-9), 82 kDa (active MMP-9), 72 kDa (pro-MMP-2), 66 kDa (intermediary form MMP-2) and 62 kDa (active MMP-2) (Fig. 1A). Among epidermal samples, those derived from HPV16 E7wt and E6E7 expressing cultures exhibited higher MMP-9 activity (both latent and active forms, *P<0.05*) (Figure 1A, 1B). For samples derived from the dermal equivalent, MMP-9 latent form was detected in all conditions. MMP-9 activity was down-regulated in samples from cultures expressing E7wt alone (*P<0.05*). However, no significant differences were detected when comparing samples from E6E7 expressing cultures with control (Figure 1B′). Importantly, MMP-9 activity detection was strictly dependant on the co-culture with keratinocytes, since its activity was undetectable in dermal equivalents cultured in the absence of an overlying epidermal component (Figure 1A). Latent, intermediate and active MMP2 forms were detected in all epidermal and dermal equivalent samples analyzed. Overall, dermal equivalent samples exhibited higher MMP-2 activity than epidermal ones. This is an expected result, since dermal equivalents are composed of type I collagen, a MMP-2 activity inducer [33]. In this case, no differences in MMP-2 activity were observed in dermal equivalents cultures in the absence of keratinocytes (Figure 1A).

We also used immunoblots to determine MMP-9 expression levels in the same organotypic cultures samples. As shown in Figure 1C, active MMP-9 protein expression was detectable in all samples. Samples from cultures expressing E7wt alone exhibited higher pro-MMP-9 levels. However, no significant differences in pro-MMP-9 were detected when comparing samples from E6E7 expressing cultures with control cells.

Our observations indicate that HPV16 E7wt induces MMP-9 activity in keratinocytes grown in a 3-D culture environment but not in monolayer cultures.

### HPV-16 E7 expression down-regulates RECK and TIMP-2 in primary HFKs

**Figure 2:**
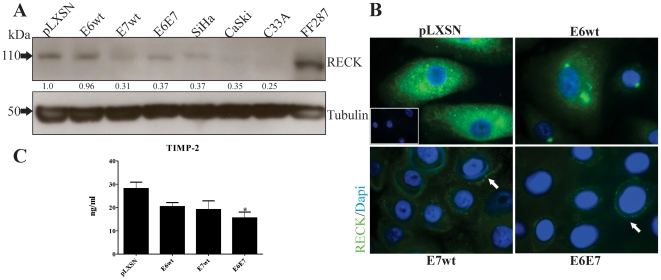
HPV16 oncoproteins down-regulate RECK and TIMP-2 in HFKs. *A,* Monolayer cultures of primary HFKs were transduced with pLXSN-based retroviral vectors expressing HPV16 E6wt and/or E7wt. RECK levels were determined by Western blot. Beta-actin was used as loading control. FF287 cell line was used as a positive control. *B,* Immunofluorescence detection of RECK (*green*) in HFKs expressing HPV oncoproteins. RECK is clearly detected in control and HPV16 E6wt expressing HFKs, while E7wt and E6E7-expressing cells exhibit reduced protein levels (*arrows*). Nuclei were counterstained with DAPI (*blue*). Original magnification 1000×. *C,* TIMP-2 was determined by ELISA (Biotrak). *P<0.05*.

The results described above indicate that HPV16 E7 induces MMP-9 expression and activity in primary keratinocytes. Therefore, we decided to analyze if this viral oncoprotein has an effect on the MMPs regulators RECK and TIMP-2. RECK is a (GPI)-anchored glycoprotein that negatively regulates matrix metalloproteinases, including MMP-9 [12], [13], [14]. RECK expression was determined by immunoblot in HFKs transduced with HPV16 oncoproteins and in three human cervical cancer-derived cell lines (SiHa, CaSki and C33A). As shown in Figure 2A, RECK was clearly detected in HFKs transduced with E6wt and control vector. Conversely, lower levels of this protein were observed in HFKs expressing HPV16 E7 or E6E7 and in cervical cancer-derived cell lines (Figure 2A).

These observations were confirmed by immunofluorescence analysis. As shown in Figure 2B, RECK was detected at high levels in pLXSN and E6wt transduced-keratinocytes but was expressed at very low levels in E7wt and E6E7 transduced-cells (Figure 2B). Strong anti-RECK signal was observed in the perinuclear region and cytoplasm of keratinocytes transduced with pLXSN and E6wt HFKs. On the other hand, HPV16 E7wt and E6E7 expressing cells exhibited a faint RECK staining that was restricted to cell membrane (Figure 2B). Interestingly, the levels of a second MMP regulator, TIMP-2, were also down-regulated in HFKs transduced with HPV16 E6E7 when compared to control cells (Figure 2C) (*P<0,05*).

**Figure 3:**
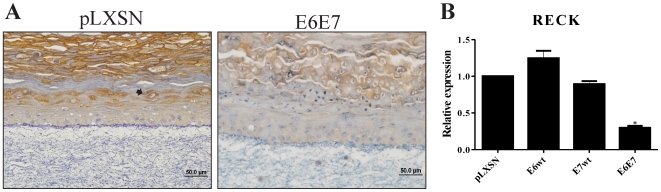
Expression of HPV-16 E6/E7 down-regulates RECK in organotypic cultures. *A,* Representative immunoreactivity of RECK in control and HPV16 E6E7 expressing rafts cultures. Note the strong RECK membrane staining pattern (black arrow) in control compared to the faint staining in HPV16 E6E7 expressing rafts cultures. *B,* Quantitative real-time PCR (qRT-PCR) of *RECK* expression in organotypic cultures. The relative expression levels were normalized to tubulin. Bars represent the means of triplicate experiments; *bars*, ±SE.

Finally, we observed that RECK expression was clearly detected in cells from the upper third of the spinous and granular layers of control organotypic cultures (Fig. 3A). On the other hand, cultures expressing E6/E7 oncogenes exhibited a faint RECK immunostaining (Fig. 3A). This result correlates with reduced RECK mRNA levels in these cultures (Fig. 3B), suggesting that the mechanism of down-regulation occurs, at least in part, at the transcriptional level. Altogether, our results indicate that HPV16 oncoproteins contribute to the down regulation of at least two different MMPs regulators.

### RECK is down-regulated in CIN III and invasive cervical carcinoma samples

The results described above prompted us to analyze if RECK down-regulation may occur *in vivo* in the context of cervical pathology. For this purpose, RECK levels were determined in cervical biopsies from patients with cervicitis (n = 16), cervical intraepithelial neoplasia grade I (CIN I) (n = 20), CIN II/III (n = 18) and invasive carcinoma (n = 12). Down-regulation of RECK was observed in nearly all invasive carcinoma (92%) and half of the (50%) CIN II/III cases. On the other hand, most of the cervicitis (87.5%) and CIN I (80%) samples exhibited strong RECK signal (Table 1).

**Table 1:** RECK expression in cervicitis, CIN I, CIN II/III and invasive cervical carcinomas.

	Cervicitis	CIN I	CIN II/III	Invasive
**Cases (%)**	16(100)	20(100)	18(100)	12(100)
**Weak** *	2(12,5)	4(20)	9(50)	11(92)
**Strong** **	14(87,5)	16(80)	9(50)	1(8)

n = 66;

* Weak = no staining or faint staining;

** Strong = moderate or strong staining.

**Figure 4:**
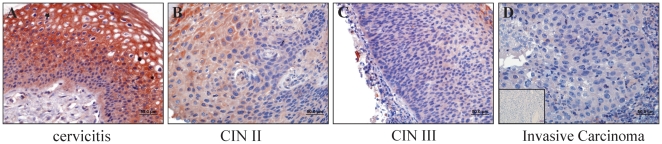
RECK is down-regulated in CIN II/III and invasive carcinoma samples. Representative immunoreactivity of RECK in (A) Cervicitis, (B) cervical intraepithelial neoplasia II (CIN II), (C) cervical intraepithelial neoplasia III (CIN III) and (D) cervical invasive carcinoma. Note the strong RECK membrane staining pattern in cervicitis (black arrow), a diminished cytoplasmastic staining in CIN II/III and a very weak RECK staining in invasive carcinoma. Inset in (D): Sample incubated in the absence of primary antibody.

Figure 4 shows a representative immunostaining for RECK in cervical samples. In cervicitis tissue, strong RECK signal was observed in the membrane and cytoplasm of most cells in all cervical epithelium layers (Fig. 4A). On the other hand, CINII/III and invasive carcinoma samples displayed weak cytoplasmastic RECK staining throughout the lesion (Fig. 4B–D).

Our observations in cervical samples show that RECK down-regulation may correlate with lesion grade. Taken together, our results suggest that inhibition of RECK expression by HPV oncogenes may play a role during cervical cancer onset/progression.

## Discussion

Deregulated MMP activity plays a critical role during epithelial carcinogenesis. In this study we show that primary human keratinocytes expressing HPV16 oncoproteins exhibit up-regulated MMP-9 activity probably due to E6 and E7 mediated down-regulation of physiological MMP inhibitors RECK and TIMP-2. We also show that RECK protein expression is down-regulated in high-grade cervical lesions and cervical cancer.

Several lines of evidence indicate that MMP-9 plays an important role in cervical tumor development. Using a murine model of cervical cancer, Pahler and co-workers showed that macrophages and neutrophils infiltrating the tumor are the source of the MMP-9 secreted into the tumor stroma [34]. Moreover, a recent study has shown that HPV-transformed cells can instruct monocytes to produce MMP-9 through a mechanism involving paracrine STAT3 activation, autocrine CCR2 stimulation and intracellular Ca^2+^ signaling [35]. Importantly, high MMP-9 expression levels and activity have been correlated with poor clinical outcome in cervical cancer patients [26]. Recently, it has been observed that the early E2 protein of HPV8 increases MMP-9 expression by direct activation of the MMP-9 promoter [36]. Besides, up-regulation of MMP-9 possibly trough activation of the MEK/ERK pathway has been detected in cells expressing HPV-18 E7 [37]. Here we show that human keratinocytes expressing HPV-16 E7 exhibit up-regulated MMP-9 activity in organotypic cultures. Since no E2 protein is expressed in our system and no differences in total MMP-9 protein levels were verified between control and E7 expressing cells we deduce that E7 mediates MMP-9 activation through an alternative pathway. These observations suggest that MMP-9 could be a common target for different HPV types, although different mechanisms and viral proteins may be involved. Nonetheless our results show that HPV-infected epithelial cells may be a significant source of MMP-9 *in vivo*, a fact that might be important in the evolution of HPV-associated lesions.

Increased MMP-9 activity may result from at least two no mutually exclusive processes: increased protein expression/stability or decreased MMP-inhibitors levels. Here we show that cells acutely transduced with HPV-16 oncoproteins display reduced levels of MMP-inhibitors TIMP-2 and RECK when grown in monolayer or organotypic cultures. On the other hand, in our settings, viral oncoproteins did not affect total MMP-9 levels.

TIMP-2 role in cervical carcinoma has been addressed in previous studies shedding inconclusive results. This inhibitor was detected as highly expressed in invasive cervical carcinoma tumor cells and stroma [38]. On the other hand, using a series of 150 cervical carcinomas and 152 CIN lesions Branca and co-workers observed that TIMP-2 is down-regulated in invasive tumors and correlated low levels of the inhibitor with poor prognosis and higher invasive potential [39]. These apparently contradictory results may reflect the unique TIMP-2 dual role as an inhibitor and activator of MMPs [9]. Here we show that TIMP-2 may be down-regulated by acute expression of HPV oncoproteins which may favor deregulated MMP activity in the context of HPV infection.

RECK is a membrane bound protein that exerts inhibitory effects on the transcription, synthesis, activation and activity of MMPs. In particular, this protein can inhibit the secretion and activity of MMP-2, MMP-9 and MT1-MMP [12], [13], [15] as well as MMP-9 transcription [14]. Down-regulation of RECK has been documented in many human cancers, including various carcinomas, and correlates with aggressiveness and poor prognosis of the disease [22], [23], [40]. We have previously shown that RECK mRNA expression is down-regulated in HPV16-positive cervical carcinoma cell lines [29]. Here, we show that these cells lines also present reduced RECK protein levels. Importantly, down-regulation of RECK was associated with HPV16 E7 expression. On the other hand, a recent report showed that HeLa cells where E6 oncogene was silenced with specific siRNA exhibited up-regulation of several anti-oncogenes including RECK [30]. These observations suggest that both oncoproteins may be involved in RECK down-regulation. Furthermore, they are in line with our results showing that, in organotypic cultures, co-expression of HPV16 E6 and E7 induce lower levels of RECK than each oncoprotein expressed individually. Characterization of RECK in human cervical specimens showed that its expression was higher in cervicitis samples than in CINII/III and invasive carcinoma samples indicating that RECK down-regulation may correlate with lesion grade. Therefore, pending further evaluations, RECK expression could be a valuable prognostic marker for cervical lesions as has been proved for other tumors [22], [23]. It is worth noting that we also detected reduced RECK levels in C33A cells, a HPV-negative cervical cancer cell line. C33A is unique among cervical cancer derived cell lines in many aspects. For instance, it is p53 mutated. Obviously, down regulation of p53 activity in this cell line is not occurring as a consequence of HPV oncoprotein expression. However, there is little doubt that E6-mediated p53 inhibition is a critical event in cervical cancer development and maintenance. An identical line of reasoning can be applied to our observations on the effect of HPV-16 E7 on RECK expression. Therefore, we believe that this result further support the notion that RECK inhibition is an important event in cervical cancer biology. Taken together, our results indicate that inhibition of RECK expression by HPV oncogenes may play a role during cervical cancer onset/progression.

Cells lacking RECK show some characteristics such as decreased spreading, altered anterior-posterior polarity, increased migration speed and decreased directional persistence in migration, which are typical of many transformed cells [41]. On the other hand, RECK over expression has been associated with actin filaments rearrangement and decreased migratory ability and invasive potential in a human glioblastoma multiforme model [42]. Although we provide data that HPV-16 oncoproteins down-regulate RECK expression in cell cultures and cervical samples, the biological consequences of low RECK protein expression in the context of HPV-related diseases remain unknown. Previous work established that TIMP-2 regulates RECK in endothelial cells. Treatment of these cells with TIMP-2 induced RECK expression through diminished Src levels, inactivation of Rac1 and activation of Rap1, resulting in a cellular migration inhibition or directional movement impairment [43], [44]. In addition, using a nasopharyngeal carcinoma cellular model Liu and co-workers showed that the Epstein-Barr-Virus latent protein 1 (LMP1) can down-regulate RECK expression, and that Sp1 binding site on RECK's promoter is essential for this effect [45]. Importantly, RECK inhibition confers an invasive and metastatic phenotype to the cell line. The interplay between HPV oncoproteins, TIMP-2 and other cellular factors such as Sp1 and Src in the regulation of RECK are among the remaining questions arising from our findings.

Our observations identify a novel property of HPV oncoproteins that may favor a pro-tumorigenic environment. First, HPV-transformed cells can influence MMP-9 expression by tumor infiltrating myeloid cells. Besides, HPV-infected cells may contribute directly to MMP-9 activity by E7-dependent up-regulation of MMP-9 levels and activity. Further local MMP-9 activation can be reached by HPV-oncoproteins mediated down-regulation of MMP-inhibitors TIMP-2 and RECK. Finally, TIMP-2 and RECK down-regulation may favor the activation of other relevant MMPs.

To our knowledge, this is the first report describing RECK down-regulation in cervical cancer and its inhibition by HPV oncoproteins in primary keratinocytes. Furthermore, the data in this study provide the first evidence that HPV-16 E7 may regulate RECK expression. All in all, our results suggest that RECK down-regulation may be important in the natural history of cervical cancer. A systematic investigation of the existence of a correlation between RECK levels, lesion grade and HPV presence in a larger set of cervical samples is currently in progress. This study will help to determine if RECK could represent a new biomarker of cervical cancer progression and a potential target for therapy.

## Supporting Information

Figure S1 **Expression of HPV16 E6E7 oncoproteins does not affect the level and activity of MMP2, MMP9 and MT1-MMP in HFKs grown in monolayer.** *A*, Primary HFKs cultured in monolayer were transduced with pLXSN-based retroviral vectors expressing HPV-16 E6wt and/or E7wt. Western blots were carried out to determine MMP-2, MMP-9 and MT1-MMP levels. Beta-actin was used as a loading control. Extracts from HT1080 cell line was used as a positive control (*n* = 3). MMP-2 (*B*) and MMP-9 (*C*) activities were analyzed by Biotrak Activity Assay. See M&M for details. Significant 2-fold activation of MMP-9 was observed in supernatants from the HFKs expressing HPV-16 E6wt when compared to control cells. However, no differences in MMPs activities were observed between HFKs expressing both HPV-16 oncoproteins and those transduced with the empty vector (pLXSN). *P<0.05*.
(TIFF)
